# Involvement of the central nervous system in relapsed T-cell lymphoma: insights from four case studies

**DOI:** 10.1016/j.lrr.2025.100524

**Published:** 2025-06-23

**Authors:** Radu Chiriac, Lucile Baseggio

**Affiliations:** Hematology Laboratory, Hospices Civils de Lyon, Centre Hospitalier Lyon Sud, Lyon, France

**Keywords:** CNS, T-cell lymphoma, Cytology, Cytometry

## Abstract

CNS involvement in T-cell lymphoma is rare, with a 2–6 % risk of relapse. This report presents four cases of CNS relapse in aggressive T-cell lymphomas, including PTCL NOS, AITL, ALCL ALK (-), and ENKTCL. Patients experienced severe neurological symptoms, elevated CSF WBC counts, and resistance to various treatment regimens, including intrathecal HD MTX, salvage chemotherapy, and immunotherapy. Median survival was 1.5–3.5 months, highlighting the poor prognosis. The findings highlight the complexities of managing CNS relapse, the evolving understanding of prophylactic strategies, and the potential for innovative, targeted therapeutic combinations to enhance outcomes for these high-risk patients.

## 1 Introduction

Central nervous system (CNS) involvement in T-cell lymphoma is rare, with only 2 % of primary CNS lymphomas being of T-cell origin and a 2 % to 6 % risk of CNS relapse in such cases [1]. Herein, we report four cases of patients diagnosed with CNS involvement at relapse, secondary to various types of T-cell lymphoma, detailing the main patient characteristics, cytological and immunological profiles, as well as their clinical courses.

## 2 Case presentation

Case 1

**Fig. 1:**
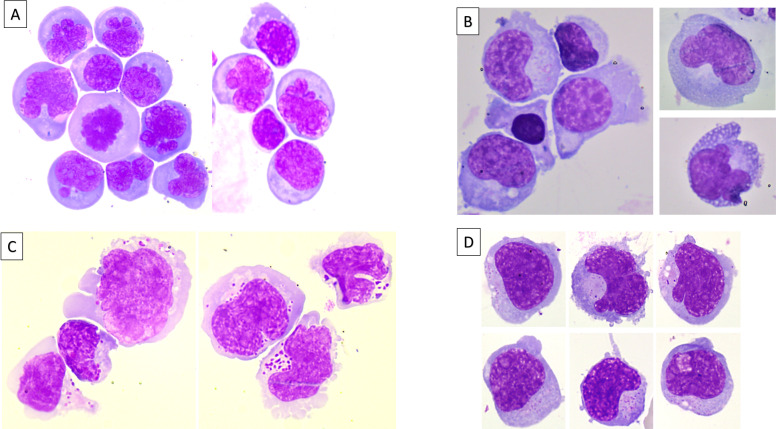
Panels A, B, C, and D, May-Grunwald Giemsa stain, × 100 objective, showing different aspects of lymphoma involvement in the CNS in PTCL-NOS, TFHL-AITL, ALCL ALK-negative, and ENKTCL, respectively.

**Fig. 2:**
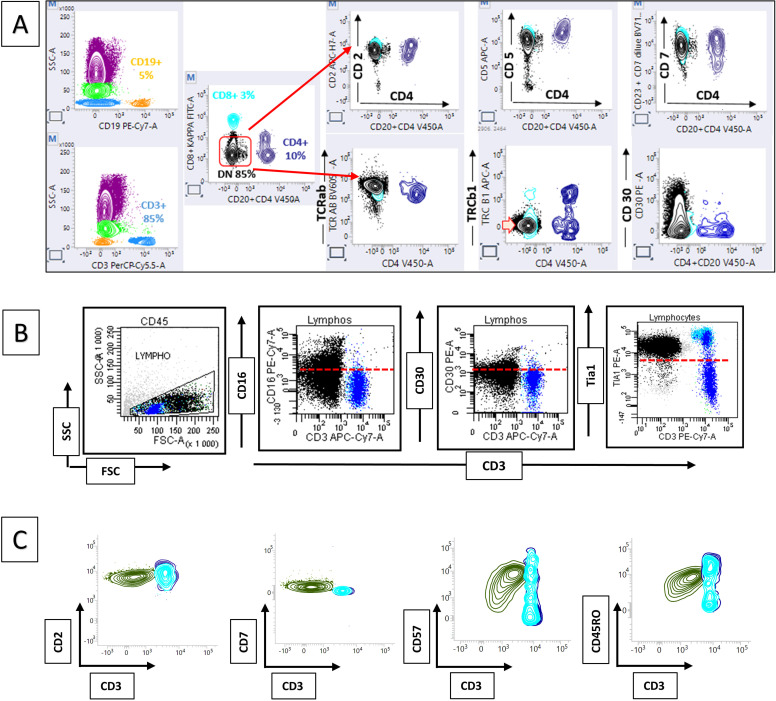
Panels A, B, and C: Flow cytometry profiles showing PTCL-NOS involvement (black plot, T-cells: CD3+, CD5+, CD4-, CD8-, expressing TCR alpha/beta), ALCL ALK-negative (black plot, CD4+ cytotoxic T-cells with strong CD30 expression), and ENKTCL (green plot, aberrant NK/T-cell phenotype: negative for CD3 and CD5, but positive for CD2, CD7, CD57, and CD45RO).

A 50-year-old man was recently diagnosed with a circulating phase of peripheral T-cell lymphoma, not otherwise specified (PTCL-NOS), belonging to the GATA-3 molecular subgroup. One year after the initial diagnosis, at the end of the sixth cycle of CHOP (cyclophosphamide, doxorubicin, vincristine, prednisone) chemotherapy, he presented with fever, dizziness, headache, nausea, and left-sided facial paralysis. No circulating lymphoma cells were detected in peripheral blood. Brain and full-spine contrast-enhanced magnetic resonance imaging (MRI) showed cauda equina meningoradiculitis and FLAIR hypersignal in the hippocampus. Lumbar puncture revealed hypercellular cerebrospinal fluid (CSF) with 800 white blood cells (WBC) per mm³ and monomorphic lymphoid cells characterized by medium size, irregular nuclear contours, condensed chromatin, and intensely basophilic cytoplasm (Fig. 1A). Flow cytometry (FCM) identified a predominant population (90 % of CSF WBCs) of CD3+, CD5+, CD4-, CD8- T-cells expressing TCR alpha/beta (Fig. 2A). Polymerase chain reaction (PCR) confirmed that the CSF population was clonally identical to the initial peripheral blood clone. The patient was treated with intrathecal high-dose methotrexate (Methotrexate) in combination with ifosfamide (IFO). Despite treatment, he experienced progressive neurological decline and died four weeks later due to disease progression and refractory seizures.

Case 2

A 60-year-old man with a one-year history of follicular helper T-cell lymphoma, angioimmunoblastic type (TFHL- AITL), undergoing CHOP chemotherapy, presented after the fifth cycle with significant weight loss (10 kg in one month), speech difficulties, and diplopia. Concomitantly, he was diagnosed with methicillin-resistant *Staphylococcus haemolyticus* endocarditis affecting the native aortic valve, characterized by an 8 × 5 mm mobile mass, for which he received six weeks of daptomycin. Brain MRI revealed multiple septic emboli and a heterogeneous periventricular intra-axial lesion with a necrotic component, raising suspicion for CNS lymphoma involvement. Lumbar puncture showed clear CSF with 61 WBCs/mm³ and a mixed cellular composition, including small lymphocytes, plasmacytoid cells, a few plasma cells, and numerous large lymphoid cells (Fig. 1B). Although FCM did not confirm a lymphoma population, molecular testing identified a clonal T-cell population matching the initial lymph node biopsy. Based on these findings, a diagnosis of isolated CNS relapse of lymphoma was made, and the patient began treatment with R-MPV-VP16 (rituximab, HD-MTX, vincristine, etoposide (Etoposide), and procarbazine). Following the first cycle, the patient developed recurrent seizures and progression of cerebral lesions. A salvage regimen combining brentuximab vedotin (BV) and bendamustine (Be) was initiated; however, he died two months later from infectious complications and repeated episodes of status epilepticus.

Case 3

A 64-year-old woman with stage IV ALK-negative anaplastic large cell lymphoma (ALCL), previously treated with six cycles of BV-CHP (brentuximab vedotin, cyclophosphamide, doxorubicin, prednisolone) and in complete remission for six months, presented with progressively worsening motor deficits and increasing gait disturbances. Electromyography indicated findings consistent with both lymphomatous infiltration and a demyelinating neuropathy, possibly related to prior anti-CD30 chemotherapy. CSF analysis revealed 500 WBCs/mm³, and cytological examination identified medium- to large-sized neoplastic cells with irregular nuclei, pale cytoplasm, and prominent azurophilic granulations (Fig. 1C). FCM confirmed the presence of CD4+ cytotoxic T-cells with strong CD30 expression, consistent with the initial lymph node biopsy, supporting the diagnosis of neuromeningeal relapse (Fig. 2B). Intrathecal HD-MTX and hydrocortisone (HCT) were administered, leading to a partial improvement in neurological symptoms. However, prior to the sixth treatment cycle, a repeat lumbar puncture revealed ongoing lymphomatous infiltration. Given the patient’s deteriorating clinical status, palliative care was initiated.

Case 4

A 55-year-old man with a history of extranodal NK/T-cell lymphoma, nasal type (ENKTCL), diagnosed one year earlier and treated with intensified therapy including autologous stem cell transplantation (ASCT), developed acute bilateral facial paralysis four months post-transplant. Clinical examination was otherwise unremarkable, and no circulating lymphoma cells were detected. Notably, plasma EBV DNA levels were markedly elevated at 470,000 copies/mL (reference: 180–500 copies/mL). Lumbar puncture revealed 250 WBCs/mm³, and cytology showed monomorphic medium- to large-sized lymphomatous cells (Fig. 1D). FCM of the CSF demonstrated an aberrant NK/T-cell phenotype, negative for CD3 and CD5 but positive for CD2, CD7, CD57, and CD45RO, confirming CNS relapse (Fig. 2C). The patient was treated with pembrolizumab (Pem) and HD-MTX, resulting in temporary symptom improvement. Unfortunately, three weeks later, he died from an acute subdural hematoma.

## 3 Discussion

This report details four clinical cases of T-cell lymphoma with CNS involvement at relapse. Each case demonstrates lymphoma relapse into the CNS despite initial treatment, involving aggressive subtypes such as PTCL NOS, TFHL-AITL, ALCL ALK-negative, and ENKTCL.

The median time from initial diagnosis to CNS relapse was 12 months, with three cases occurring during or within 6 months of frontline therapy completion. All patients experienced isolated CNS relapse, with mixed leptomeningeal and parenchymal involvement in the first two cases and only leptomeningeal involvement in the last two. Median overall survival was 1.5 months for patients with mixed involvement and 3.5 months for those with leptomeningeal involvement alone.

Despite efforts to identify patients at risk of CNS relapse, the role of prophylactic therapy remains controversial [1]. None of the cases presented received prophylactic treatment. Chemoprophylaxis has been less studied in T-cell lymphoma, and the 2022 National Comprehensive Cancer Network guidelines do not routinely recommend CNS evaluation unless symptoms are present [2]. A 2021 presentation at the ASH Annual Meeting reported on a cohort of 83 patients with T-cell lymphoma who experienced CNS progression/relapse; the study found that CNS prophylaxis, administered to 17 patients, did not significantly alter the time to CNS involvement [3].

In a recent study by Bhansali et al. [4], CNS-directed prophylaxis was administered in only 16 % of cases and showed no clear benefit. Frontline therapies, including anthracyclines and (Etoposide), did not reduce the risk of CNS relapse. Hematopoietic cell transplantation in first complete remission was associated with a significant reduction in CNS relapse (HR = 0.38; *P* = .018), although this effect was not consistently observed in high-risk subgroups. The CNS relapse in T-cell lymphoma index (CITI) is a validated risk model that incorporates clinical and histologic features at diagnosis to stratify patients by CNS relapse risk and may help identify those who could benefit from targeted prophylaxis.

Elevated WBC counts in the CSF were consistently observed across all cases, indicating significant inflammatory or neoplastic activity in the CNS, a common feature of lymphomatous meningitis or CNS lymphoma involvement [5]. FCM in these cases revealed key immunologic profiles, notably the aberrant expression of T-cell markers (CD3, CD5, CD4, CD8) and NK-cell markers (CD57). While FCM was valuable in detecting clonal lymphoid populations, it has limitations, as seen in Case 2, where a clear lymphoma population was not identified. This underscores the need for PCR when WBC counts are low or lymphoma markers are absent.

Outcomes for patients with CNS progression or relapse are poor, with few studies reporting a median overall survival exceeding 6 months, likely due to concurrent systemic progression and an aggressive disease phenotype ([3,6]). Currently, there is no standard of care for these patients. Yi et al. studied 20 patients, with eight undergoing radiation, nine intrathecal therapy, and eight systemic therapies; eight patients had a combination of at least two treatments [7]. A complete CNS response was observed in 45 % (*n* = 9), and among three patients who had ASCT, one survived an additional 8.3 years. The Czech group reported that eight patients with CNS relapse received HD-MTX, often combined with agents like thiotepa [8]. Intrathecal treatment was rarely used. Salvage therapies included platinum-based regimens (*n* = 5), whole brain radiation (*n* = 5), and BV with gemcitabine (*n* = 1). Of the 16 patients treated for CNS relapse, only two achieved a complete response: one after ASCT and one after whole brain radiation plus ASCT.

In case 1, intrathecal HD MTX, and IFO failed, leading to rapid disease progression and death from seizures. In case 2, despite salvage therapy with rituximab, MTX, BV, and Be, the patient experienced recurrent seizures, disease progression, and died from infections and status epilepticus. In case 3, intrathecal HD-MTX, and HCT initially improved symptoms, but new lymphomatous infiltration led to a shift to palliative care. In case 4, Pem and HD-MTX caused symptom regression, but the patient ultimately died from an acute subdural hematoma.

CNS involvement in mature T- and NK-cell neoplasms appears to be more prevalent in specific histologic subtypes and clinical contexts. PTCL, NOS, is the most frequent subtype associated with CNS relapse, accounting for 25 % of cases; ENKTCL, though less common in North American cohorts, is a well-established risk subtype in endemic regions [4]. Additionally, the presence of two or more extranodal sites, including bone marrow or peripheral blood involvement, significantly increases the risk of CNS relapse [4]. In our case series, however, only two patients (Case 1 and Case 3) demonstrated involvement of both bone marrow and peripheral blood at initial diagnosis.

Fig. 1,2,Table 1

**Table 1:** Clinical features, treatment and outcome at relapse.

Case	Age/Sex	Diagnosis	Time to CNS Involvement	Treatment at CNS Relapse	Duration of CNS Involvement	Outcome
1	50/M	PTCL-NOS (GATA-3 subtype)	12 months (end of 6th CHOP cycle)	Intrathecal high-dose MTX + IFO	1 month	Died from disease progression and refractory seizures
2	60/M	TFHL, AITL	11–12 months (after 5th CHOP cycle)	R-MPV-VP16; then BV + Be	2 months	Died from infections and status epilepticus
3	64/F	ALCL, ALK-negative	12 months (6 months after remission)	Intrathecal high-dose MTX + HCT	2 months	Disease progression → palliative care
4	55/M	ENKTCL, nasal type	12–13 months (4 months post-ASCT)	Anti PD-1 + high-dose MTX	1 month	Died from acute subdural hematoma

Abbreviations: CHOP (cyclophosphamide, doxorubicin, vincristine, prednisone); MTX (methotrexate); IFO (ifosfamide); R-MPV-VP16 (rituximab, methotrexate (Methotrexate), procarbazine, vincristine, etoposide); BV (brentuximab vedotin); Be (bendamustine); HCT (hydrocortisone); ASCT (autologous stem cell transplantation); VP-16 (etoposide); PD-1 (programmed cell death protein 1).

Despite the variety of treatment approaches, the overall prognosis remained poor, with all patients ultimately succumbing to their disease, whether due to progression, treatment complications, or infections. This highlights the unpredictable and complex nature of complications in these cases, further complicating efforts to improve patient outcomes.

## 4 Conclusion

CNS involvement in T-cell lymphoma, though rare, is a significant complication with a poor prognosis, often manifesting with distinct morphological features. Early neurological symptoms should prompt immediate investigation for CNS infiltration, which can present with characteristic changes in CSF, such as elevated white blood cell counts. These cases highlight recurring challenges in aggressive T-cell and NK/T-cell lymphomas, including the morphological presentation of CNS relapse and resistance to treatment. Given the limited success of single-agent therapies in altering the morphological progression of the disease, exploring combination strategies with targeted treatments for relapsed or refractory T-cell lymphoma may offer promising therapeutic potential.

## Ethics approval statement

The study was approved by Biological Resource Centre policy of the Hospices Civils de Lyon. The procedures followed were in accordance with the Declaration of Helsinki. The protocol received approval from the institutional review board (number: 24–5367)

## Patient consent statement

Informed consent was obtained from all the patients.

## Permission to reproduce material from other sources

The authors declare no use of third-party material in this study for which formal permission is required

## Clinical trial registration

Not applicable

## Funding statement

This research received no specific grant from any funding agency in the public, commercial, or not-for-profit sectors.

## Data availability

Data sharing is not applicable to this article as no new data were created or analysed in this study.

## CRediT authorship contribution statement

**Radu Chiriac:** Writing – original draft, Conceptualization. **Lucile Baseggio:** Resources, Investigation.

## Declaration of competing interest

The authors declare that they have no known competing financial interests or personal relationships that could have appeared to influence the work reported in this paper.
